# Mycophenolate Mofetil-Related Enterocolitis and Weight Loss: A Pediatric Case Series

**DOI:** 10.1155/2012/624168

**Published:** 2012-10-23

**Authors:** Dana M. H. Dykes, Sean R. Moore, D. Brent Polk, Michael J. Rosen, Marcia L. Wills, Brian Morris, Jeanine S. Maclin, Janaina Nogueira, Avi Katz, Tracey E. Hunley, Judith Pugh, Shehzad Saeed

**Affiliations:** ^1^Division of Gastroenterology Hepatology and Nutrition, Cincinnati Children's, 3333 Burnet Avenue MLC 2010, Cincinnati, OH 45229-3039, USA; ^2^Division of Gastroenterology, University of Southern California, 4650 Sunset Boulevard, MS #126, Los Angeles, CA 90027, USA; ^3^Division of Gastroenterology, Hepatology and Nutrition, Department of Pediatrics, Vanderbilt University School of Medicine, 2200 Children's Way, 11th Floor, Doctors' Office Tower, Nashville, TN 37232-2576, USA; ^4^Seacoast Pathology, Aurora Diagnostics, 1 Hampton Road, Suite 208, Exeter, NH 03833-4849, USA; ^5^Division of Gastroenterology, Oschner Children's Health Center, 1315 Jefferson Highway, New Orleans, LA 70121, USA; ^6^Division of Pediatric Gastroenterology and Nutrition Sciences, University of Alabama at Birmingham, 1600 7th Avenue. S., Lowder Blgd., Ste. 618, Birmingham, AL 35233, USA; ^7^Department of Nephrology, University of Minnesota, Pediatric Specialty Care Discovery Clinic, 2512 Building, 2512 S. 7th Street, Minneapolis, MN 55454, USA; ^8^Division of Pediatric Nephrology, Vanderbilt University School of Medicine, 2200 Children's Way, 11th Floor, Doctors' Office Tower, Nashville, TN 37232, USA; ^9^Department of Pathology, University of Arizona, 1501 N. Campbell Avenue, P.O. Box 245108, Tucson, AZ 85724-5108, USA

## Abstract

Mycophenolate mofetil (MMF) is an immunosuppressive medication utilized in the management of both autoimmune and solid organ transplant patients. Diarrhea is a common gastrointestinal side effect of MMF, but more severe forms of GI symptoms are described in renal transplant patients with a distinct pattern of histopathologic change, similar to graft-versus-host disease or Crohn's disease. This rare entity, commonly referred to as “MMF-related enterocolitis,” has been described in adult patients, mostly in renal transplant patients, and in only two pediatric renal transplant patients. In previously reported cases, symptoms and abnormal histopathology improve with dose reduction of MMF. We describe a series of three pediatric patients with varied underlying disease process who presented with severe diarrhea and histopathologic findings characteristic of MMF-related enterocolitis, who share a novel finding of weight loss as a complication of MMF-related enterocolitis in pediatric patients.

## 1. Introduction

Mycophenolate mofetil (MMF, Cellcept Genentech, San Francisco, CA) is an oral immunosuppressive agent commonly prescribed in the United States to prevent rejection in solid organ transplant recipients and to manage select autoimmune diseases, both in adults and children.

The active metabolite of MMF is mycophenolic acid (MPA), a potent inhibitor of inosine 5′-monophosphate dehydrogenase type II (IMP-2), a critical enzyme in the *de novo *purine synthesis pathway of B and T lymphocytes [1]. This inhibition results in cell cycle arrest, thereby interrupting production of B and T lymphocytes [2]. The most common side effects of MMF are related to bone marrow and gastrointestinal toxicity, typically diarrhea [3]. Patients who have had symptoms severe enough to necessitate endoscopic evaluation have demonstrated abnormal histopathologic findings which may mimic infection, acute graft-versus-host disease, or Crohn's enterocolitis and are characterized by crypt cell apoptosis and small bowel villous atrophy [4]. This entity, described as “MMF-related enterocolitis” has been well described in adults, but is so far described in only two pediatric renal transplant recipients presenting with diarrhea and abdominal pain [5]. We describe a novel presentation of MMF-related enterocolitis in two pediatric solid organ transplant recipients and an adolescent with Wegener's Granulomatosis in whom patients presented with severe diarrhea, weight loss, and, in one patient, growth failure. The enterocolitis in these three cases share common histologic features, suggesting that the effect of MMF is independent of the underlying disease process. We discuss the implications for diagnosis and treatment of MMF-induced diarrhea in such patients.

## 2. Case Presentation

**Case 1**

A 17-year-old female patient was admitted with acute renal allograft rejection, nonbloody diarrhea for one month, and vomiting for two weeks. She had also experienced a six-pound weight loss during this time period and tenesmus. The patient had received a living unrelated renal transplant for an end stage renal disease of unknown etiology almost 2 years prior to this admission. Initial immunosuppression consisted of induction with basiliximab (Simulect Novartis Pharmaceuticals, East Hanover, NJ), sirolimus (SRL, Rapamycin, Rapamune, Pfizer, New York City, NY), and tacrolimus (Prograf Astellas Pharma US, Deerfield, IL). Shortly after transplantation, her tacrolimus was discontinued secondary to thrombotic microangiopathy, and mycophenolate mofetil was started. Two months prior to admission, her MMF dose was increased to 1000 mg twice daily (1330 mg/m^2^/day) and sirolimus to 2 mg orally twice daily (2.6 mg/m^2^/day) for two concurrent rejection episodes.

At the time of this admission, the patient was afebrile and had a normal physical examination except for visible surgical scars and bruising from multiple IV attempts. Her home medications were restarted upon admission and included low dose daily prednisone 5 mg/d, sirolimus (3 mg/day), mycophenolate mofetil (2000 mg/day given twice daily), darbepoetin, macrodantin, atorvastatin, ethinyl estradiol/levonorgestrel oral contraceptive pills, iron polysaccharide, ranitidine, and sodium bicarbonate. Admission laboratory studies were normal with the exception of mild anemia with a hemoglobin of 10 g/dL (normal 12–16 g/dL), cystatin C of 2.8 mg/L which estimated the patient's GFR at 29%, creatinine of 5.1 mg/dL, and a normal C-reactive protein. 

**Figure 1:**
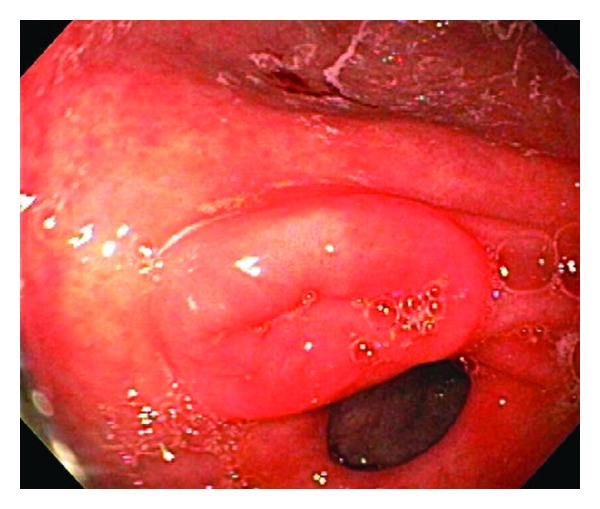
Umbilicated mass at the pylorus.

**Figure 2:**
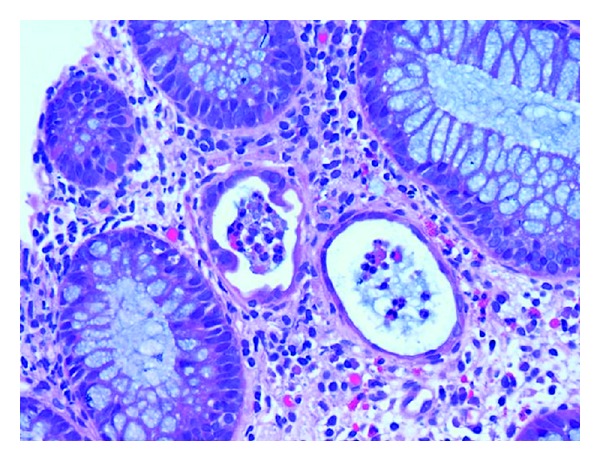
Colonic biopsy from solid organ transplant patient. Sections of the mucosa show interspersed crypts with dilatation lined by flattened epithelium with inflammatory abscess. The cellularity of the surrounding lamina propria is increased (Hematoxylin and Eosin 40×).

 Evaluation for infectious etiology revealed a negative work-up for cytomegalovirus in the stool, fecal white blood cells, cryptosporidium, *C. difficile*, Giardia, ova and parasites, enterovirus (by stool PCR), parvovirus (by stool PCR), rotavirus, and stool bacterial culture. No fecal reducing substances or occult blood were detected. Due to the persistence of symptoms the patient underwent evaluation with esophagogastroduodenoscopy (EGD) and flexible sigmoidoscopy. Grossly, the patient had an umbilicated mass in the pylorus (Figure 1) that resembled a pancreatic rest, but otherwise both EGD and flexible sigmoidoscopy were visually normal. Histopathologic samples from the sigmoid colon biopsy revealed significant abnormalities including tubular crypts with mild focal distortion with interspersed dilated crypts lined with flattened epithelial cells, intraluminal cellular debris and apoptotic cells (Figure 2). There were increased lymphocytes and eosinophils in the lamina propria surrounding damaged crypts. With no identified infection to explain the abnormal histopathology, diarrhea, and weight loss, the biopsies were felt to be consistent with previously reported cases of MMF-related enterocolitis. The patient improved after dose reduction of MMF and was discharged home on hospital day 10.

**Case 2**

An 8-year-old male, status postorthotopic liver transplantation for biliary atresia one year prior to presentation was admitted with a history of diarrhea for the last few weeks. He had also experienced an 11 pound unexplained weight loss over the last three months. Diarrhea was nonbloody but voluminous and totaled almost 2 liters per day. The patient denied any fever or vomiting but had decreased appetite. The patient's initial immunosuppression after transplantation consisted of mycophenolate mofetil, tacrolimus, and low dose prednisone. He had one episode of rejection 3 months prior to admission that had been treated with prednisone with good response.

On admission the patient was clinically dehydrated with a nongap metabolic acidosis. He was noted to have an otitis media and was treated with ceftriaxone for 2 days. His baseline immunosuppression was accomplished with mycophenolate mofetil 750 mg twice daily (1600 mg/m^2^/day), tacrolimus 4 mg every morning and 5 mg every evening (9.7 mg/m^2^/day or 0.36 mg/kg/day), and prednisone 10 mg every night (2.5 mg/kg/day) was continued along with his methylphenidate and lansoprazole. Other than slightly low potassium of 3.0 mmol/L and a bicarbonate of 18 mcmol/L, admission labs were normal. The patient continued to have voluminous diarrhea after admission requiring fluid replacements with bicarbonate. 

Infectious work-up was negative for *C. difficile* toxin, rotavirus, ova and parasites, Giardia and Cryptosporidium, fecal white blood cells, and bacterial stool culture. Blood testing for CMV and EBV by PCR was also negative. EGD and colonoscopy were performed after diarrhea, and poor weight gain persisted during the first days of hospitalization. While the tissue was visually normal, microscopic examination of the colon revealed scattered apoptosis, crypt distortion, flattened epithelium, and cellular debris within the glands. Chronic inflammatory cells with prominent eosinophils and rare neutrophils were present in the surrounding lamina propria. Due to concern of MMF-related enterocolitis, the medication was discontinued with resultant improvement in stool frequency and consistency within 2 weeks with no other intervention.

**Case 3**

A 14-year-old male with Wegener's Granulomatosis was diagnosed at 8 years of age after presenting with pulmonary hemorrhage. At that time, he underwent percutaneous renal biopsy, which showed pauci-immune sclerosing crescentic glomerulonephritis, consistent with Wegener's Granulomatosis. He was also found to be p-ANCA positive.

The patient was initially treated with methotrexate for the first three years after diagnosis, but due to rising aminotransferase levels, methotrexate was transitioned to azathioprine. Aminotransferase levels improved but did not normalize on low dose azathioprine, so the patient was ultimately transitioned to MMF. The patient had been on MMF for the past two years, but the dose had been gradually increased from 500 mg orally every morning and 250 mg every evening (total daily dose of 650 mg/m^2^/day) to 1 g twice daily (3000 mg/m^2^/day) due to active Wegener's Granulomatosis (worsening microscopic hematuria, urinary sediment, and increased erythrocyte sedimentation rate). In the weeks after beginning high dose MMF, he developed nonbloody diarrhea, which initially responded to a decrease in dietary fiber and an increase in dosing frequency (500 mg of MMF four times daily). 

 At age 14 years, he was admitted with a 3-week history of nonbloody diarrhea, poor weight gain over the past months since starting high-dose MMF, and more acutely a 26 pound weight loss over the last few months. He also had stunting of his linear growth that had been attributed to corticosteroid use. He reported poor appetite and early satiety, fatigue, and two episodes of nonbloody, nonbilious emesis. In addition to high-dose MMF, his home medications included losartan, ferrous sulfate, darbepoetin, omeprazole, famotidine, a multivitamin, and trimethoprim/sulfamethoxazole for PCP prophylaxis.

 On physical exam, he was comfortable but was cachectic with prominent ribs and wasted thin extremities and musculature. His exam was notable for a soft, nontender abdomen. Review of his growth curve revealed a steady decrease in weight starting at age 12 with the introduction of MMF (see digital image in Supplimentary Material available online at doi:10.1155/2012/624168).

 Initial laboratory examinations were significant for hemoglobin of 10.0 g/dL and platelet count of 508,000/uL. Sodium, chloride, and bicarbonate were low with values of 129 meq/L, 97 meq/L, and 13 mmol/dL, respectively. Blood urea nitrogen was elevated at 32 mg/dL, and creatinine elevated at 1.2 mg/dL. Anion gap was elevated at 19. Prealbumin was low at 16 mg/dL, but liver enzymes, total protein, and albumin were normal. Thyroid studies, micronutrient levels, and C-reactive protein were all normal. Stool infectious studies were all negative. His urinalysis was notable for a specific gravity of 1.027, pH 5.5, trace albumin, moderate ketones, and 1 to 3 hyaline casts. A urine cytomegalovirus antigen was negative. His chest X-ray was normal, and a chest CT to evaluate for pulmonary hemorrhage was also normal. To correct his hyponatremic dehydration and anion gap metabolic acidosis, he was given slow intravenous hydration. 

**Figure 3:**
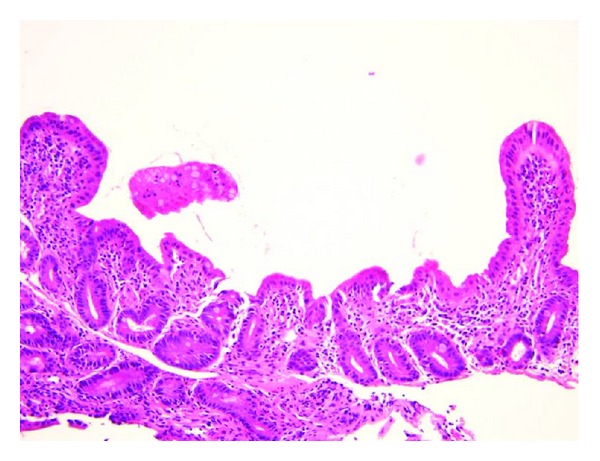
Biopsy of duodenum showing patchy villous blunting, increased intraepithelial lymphocytes, gastric mucin cell (foveolar) metaplasia, and reactive glandular changes within the crypts. No evidence of vasculitis or granuloma (Hematoxylin and Eosin 20×).

In the absence of obvious infection causing the diarrhea and failure to thrive, gastrointestinal endoscopic evaluation was performed to evaluate for other causes including active Wegener's with gastrointestinal involvement, autoimmune enteropathy, inflammatory bowel disease, infection, or other enteropathies. Visually, the duodenum and ileum appeared flattened with patches of nodularity. The rectal and sigmoid mucosa showed patches of erythematous and slightly edematous tissue with intervening areas of normal-appearing mucosa. Duodenal biopsies (Figure 3) showed small intestinal mucosa with patchy villous blunting, increased intraepithelial lymphocytes, foveolar metaplasia, and reactive epithelial changes within the crypts. Biopsies of the terminal ileum showed small bowel mucosa with pyloric metaplasia, marked villous blunting, mildly increased intraepithelial lymphocytes, and occasional acute cryptitis with focal extension of the acute inflammation into superficial mucosa. There was crypt distortion with crypt dropout and no evidence for vasculitis or granuloma formation. Biopsies from the rectosigmoid region were also abnormal and showed colonic mucosa with focal cryptitis and crypt abscesses, crypt distortion with crypt dropout, and associated crypt reactive changes with no evidence of vasculitis or granuloma formation.

Upon initial interpretation of biopsy results, celiac disease was felt to be a possibility, but since total IgA and IgG were low, negative celiac serologic testing proved difficult to interpret. Genetic testing revealed that he was negative for HLA DQ2 and DQ8 markers, so celiac disease was felt to be unlikely since these haplotypes are typically present in individuals with celiac disease [6]. With no evidence for infection, the possibility of MMF-related enterocolitis was felt to be the most likely explanation for his symptoms. 

 His MMF was changed to enteric-coated mycophenolate sodium (EC-MPS, Myfortic Novartis Pharmaceuticals, East Hanover, NJ) and a nasogastric tube was placed for gavage feedings with a gluten-free formula but was allowed to eat a normal (gluten containing) diet by mouth. His diarrhea resolved on EC-MPS, and he was tolerating full caloric goals with a combination of oral and nasogastric feeds. He was gaining weight by discharge on hospital day eight.

 He continued to receive nasogastric feeds at home over 2 months and exhibited impressive catch-up growth (Supplemental Digital Image), but still remained below the 3%ile. EGD and colonoscopy were repeated 77 days after his initial endoscopies, and all biopsies were now normal with regeneration of the villi. He continues to have excellent weight gain and is doing well with the enteric-coated MMF.

## 3. Discussion

Pediatric patients such as the ones described in this series with MMF-related enterocolitis have received little attention in the pediatric literature, yet they may represent a larger population of children on the medication with mucosal injury. The three pediatric patients described above exhibited features of MMF-related enterocolitis, including watery diarrhea without blood or mucous, along with negative work-ups for another etiology, particularly infection [4]. The severe diarrhea, weight loss, and dehydration reported in these patients necessitated endoscopic evaluation and revealed histopathologic findings similar to previously reported in other cases of MMF-related enterocolitis in adults. These findings included apoptotic cells (not typically seen in normal colonic mucosa), crypt distortion including dilated crypts lined by injured epithelium, and inflammatory changes associated with these damaged areas [4]. In adults, MMF-related pathologic changes have been described as “Crohn's disease-like” by some due to the crypt distortion, flattened epithelium, and mild inflammatory changes [7]. 

The patients described in this series illustrate a few key differences from the previously reported pediatric patients with MMF-related enterocolitis. First, the two previously reported pediatric cases both were renal transplant patients [5]. While MMF-related enterocolitis is primarily described in adult renal transplant patients, this entity has been reported in patients with other solid organ transplants, ulcerative colitis, and mixed connective tissue disease [8–10]. Our cohort of patients with varied clinical background suggests that these findings occur outside of the renal transplant population in pediatric patients as well. In addition to having different clinical backgrounds from previously reported pediatric cases of MMF-related enterocolitis, our patients were different from previously described pediatric patients in that weight loss was a predominant symptom. While immunosuppressed patients may have many explanations for weight loss and growth failure, this may represent an underrecognized complication of MMF toxicity in this at risk group. Histopathologically, the patient described in Case 3 had villous atrophy, a reported gastrointestinal side effect of MMF in adults that has not previously been described in children [11, 12]. This child was also unique in that there were several areas of gastric mucin cell (foveolar) metaplasia seen on small intestinal biopsy specimens, a complication of MMF that has not previously been reported in children and only reported from colonic biopsies in adults [13]. Unfortunately, only one patient described in this series had repeat histologic examination, but the rapid improvement with change in MMF dose is consistent with previously reported cases and supports a diagnosis of MMF-related enterocolitis. These new pediatric cases reproduce the findings in previously reported patients and also introduce new clinical associations and histopathologic findings that may be associated with MMF-related enterocolitis. 

Typically, MMF-related colitis is symptomatically improved by a 50% reduction in mycophenolate mofetil dosage [4]. Because of frequent rejection episodes, the patient described in Case 1 underwent only a 25% reduction of the MMF dose, but fortunately experienced clinical improvement (though not complete resolution) of symptoms.  Case 2 was taken off of MMF completely and subsequently improved. The patient described in Case 3 demonstrated improvements in both clinical symptoms and mucosal architecture upon changing his medication to enteric-coated mycophenolate sodium. This formulation of the drug seems to have fewer gastrointestinal side effects but has been shown to deliver equivalent levels of MPA [14]. Although it has not been explicitly described, it is possible that the delayed exposure of the drug to the intestinal epithelium helps protect against MMF-related enterocolitis [14]. 

Patients such as those described in this case series represent a high-risk group of patients with previous rejection or drug toxicity and in whom changes in immunosuppressant regimen should not be considered unless other absolutely necessary. In particular, other explanations for symptoms such as infection, graft-versus-host disease, inflammatory bowel disease, celiac disease, or other enteropathies should be explored. Solid organ transplant patients taking MMF are at high risk for infections causing diarrhea, particularly infections such as CMV that may benefit from reduced immunosuppression, and an unrecognized infection may explain the improvement in symptoms with drug withdrawal [8, 11, 15]. Despite the high rate of infections associated with diarrhea and MMF, MMF-related enterocolitis appears to be a separate entity described in up to 40% of patients taking MMF without infection or other explanation for their symptoms [11]. After appropriate evaluation for other causes, MMF-related enterocolitis should be considered as a treatable cause of severe diarrhea in a pediatric transplant patient taking the drug. 

## 4. Conclusion

Mycophenolate mofetil-related enterocolitis should be considered as part of the differential diagnosis of chronic diarrhea and weight loss in patients taking the drug, regardless of the underlying reason for immunosuppression. The diagnosis can be made based on characteristic histopathologic changes evident in the gastrointestinal epithelium and exclusion of other etiologies, such as infection. Treatment of the disorder requires dose reduction and should be undertaken cautiously.

## Supplementary Material

This graphic demonstrates the weight-for-age percentiles in an adolescent with Wegener's granulomatosis and mycophenolate mofetil (MMF)-induced villous atrophy. (A) Patient diagnosed with Wegener's at age 8 years old. (B) At age 12, began MMF with a gradual increase in dosage . (C) Hospitalized at age 14 with severe diarrhea and failure to thrive. MMF is switched to enteric coated mycophenolate sodium and nasogastric supplemental feeds initiated. (D) Nasogastric feeds discontinued.
