# Supplementary material for: Mycophenolate Mofetil-Related Enterocolitis and Weight Loss: A Pediatric Case Series
Source: Case Rep Pediatr. 2012 Oct 23;2012:624168. doi: 10.1155/2012/624168 (PMC3485763; doi:10.1155/2012/624168)

**Supplemental Image 1:** Weight-for-age percentiles in an adolescent with Wegener’s granulomatosis and mycophenolate mofetil (MMF)-induced villous atrophy. (A) Patient diagnosed with Wegener’s at age 8 years old. (B) At age 12, began MMF with a gradual increase in dosage . (C) Hospitalized at age 14 with severe diarrhea and failure to thrive. MMF is switched to enteric coated mycophenolate sodium and nasogastric supplemental feeds initiated. (D) Nasogastric feeds discontinued.


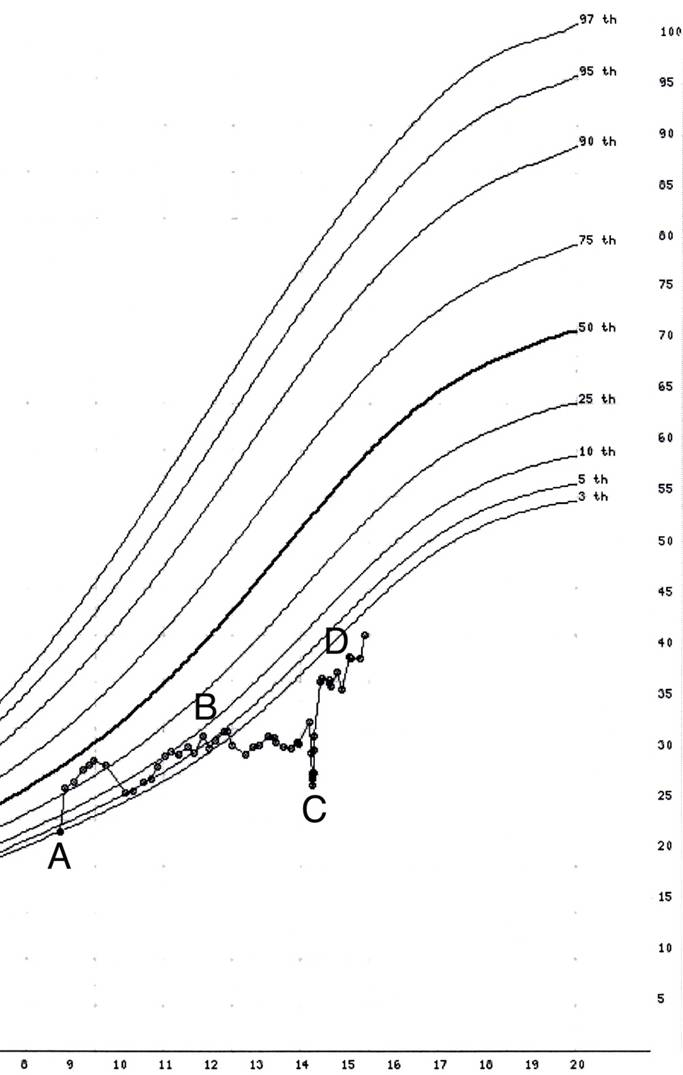

Supplement: Supplementary file 1 — This graphic demonstrates the weight-for-age percentiles in an adolescent with Wegener's granulomatosis and mycophenolate mofetil (MMF)-induced villous atrophy. (A) Patient diagnosed with Wegener's at age 8 years old. (B) At age 12, began MMF with a gradual increase in dosage . (C) Hospitalized at age 14 with severe diarrhea and failure to thrive. MMF is switched to enteric coated mycophenolate sodium and nasogastric supplemental feeds initiated. (D) Nasogastric feeds discontinued. [file 624168.f1.doc]
